# Assessing and Screening of Female Fertility in Artificially Bred Asian Yellow Pond Turtles (*Mauremys mutica*) Based on Parentage Assignment

**DOI:** 10.3390/ani14030479

**Published:** 2024-02-01

**Authors:** Xincheng Zhang, Jian Zhao, Chenyao Zhou, Wei Li, Yihui Liu, Chengqing Wei, Yakun Wang, Xiaoyou Hong, Xinping Zhu, Xiaoli Liu

**Affiliations:** 1 Key Laboratory of Tropical and Subtropical Fishery Resources Application and Cultivation, Ministry of Agriculture and Rural Affairs, Pearl River Fisheries Research Institute, Chinese Academy of Fishery Sciences, Guangzhou 510380, China; zhangxincheng2012@126.com (X.Z.); 15914516837@163.com (J.Z.); zhouchenyao@zjou.edu.cn (C.Z.); liwei@prfri.ac.cn (W.L.); 13922235385@163.com (Y.L.); wcq1970@163.com (C.W.); wykzkyky@163.com (Y.W.); hongxiaoyou1216@163.com (X.H.); 2 School of Fishery, Zhejiang Ocean University, Zhoushan 316000, China

**Keywords:** female fecundity, *Mauremys mutica*, microsatellite, breeder contributions, genetic variability

## Abstract

**Simple Summary:**

The Asian yellow pond turtle (*Mauremys mutica*) is widely traded in China, and its artificial breeding has now become a major industry. However, the insufficient offspring supply and reproductive decline of farmed turtles make wild turtles more vulnerable. The present study was mainly designed to quantify the fecundity of *M. mutica* and attempt to screen for good reproductive performance in females. The parent–offspring relationships of all offspring in four consecutive years were confirmed using sixteen microsatellite loci. We also summarised the reproductive results of all females and counted the annual number of offspring and the variation in the number of offspring. The females were then divided into three types (stable, undulating and levelling off) according to the continuity. This study can provide the basis and materials for the creation of a good reproductive group and the study of the reproductive biology of turtles in *M. mutica* aquaculture.

**Abstract:**

The Asian yellow pond turtle (*Mauremys mutica*) is widely traded in China, and its artificial breeding has now become a major industry. However, the insufficient offspring supply and reproductive decline of farmed turtles make the wild turtles more vulnerable. The present study was mainly designed to quantify the fecundity of *M. mutica* and attempt to screen for good reproductive performance in females. The genetic variability of the population and its genetic structure were also analysed. The parent–offspring relationships of all offspring in four consecutive years were confirmed using sixteen microsatellite loci. The genetic variability between the parents and offspring was low, and offspring of different years also showed little variability. We summarised the reproductive results of all females and counted the annual number of offspring and the variation in the number of offspring. The females were then divided into three types (stable, undulating and levelling off) according to the continuity. We selected seven females with good reproductive ability, which provided 16.94% of the annual contributions, while there were two females that had no offspring in four years. We also analysed the possible reasons for this difference and the importance of carrying out a family survey. This research can provide the basis and materials for the creation of a good reproductive group and the study of the reproductive biology of turtles in *M. mutica* aquaculture.

## 1. Introduction

The Asian yellow pond turtle (*Mauremys mutica*) is widely distributed in southern and central China, Vietnam and Japan [1]. This Asian freshwater turtle was once threatened with extinction, mainly due to its high demand for food, traditional medicine and as a pet [2]. Its high value makes the Asian yellow pond turtle a commonly traded turtle in China [3] and this contributes to significant illegal smuggling from Vietnam [4]. Due to their high value and large demand, the wild population is declining. Asian yellow pond turtles are also affected by habitat destruction, water pollution and invasion by alien species. The wild *M. mutica* has been listed as vulnerable on the IUCN Red List (http://www.iucnredlist.org/, accessed on 3 May 2022) and CITES Appendix II (https://cites.org/eng/app/appendices.php, accessed on 3 May 2022) since 2000. For conservation, and to meet the market demand, with the breakthrough of aquaculture technology, the Asian yellow pond turtle has been farmed as a new aquatic species in China since the 1990s, and the annual farming output of the Guangdong Province is more than 4.5 million turtles.

Fecundity refers to the ability of females to produce eggs and males to inseminate [5]. Fecundity is an important component of physical fitness in spawning-type animals [6]. Nutrition [7], living environment [8,9], population density [10], age [11] and genetic determinants [12] are the main factors influencing their fertility. Under conditions of artificial breeding, nutrition and management, variation in fecundity is largely influenced by individual genetics. However, due to its polygenic architecture, little is known about the molecular basis of the genetic mapping of fecundity. In chickens, quantitative trait locus mapping in advanced intercrosses of wild and domestic chickens, combined with expression quantitative trait locus mapping in the same birds, has been used to screen several putative quantitative trait genes for egg traits that may have been influenced by regulatory variants during chicken domestication [6]. In livestock, the method of increasing the number of effective carriers by introducing a fertility mutation into non-carrier conventional flocks based on a marker-assisted breeding programme contributes to the spread of reproductive genes and improving livelihoods in communities rearing Barbary sheep [13].

Recent studies on turtle reproduction have mainly focused on reproductive biology and mating behaviour [14,15,16], and no research has been reported on building a high-fertility population. For the Asian yellow pond turtle, breeding under normal conditions requires at least 5 years for juveniles to reach sexual maturity [17]. They are also less fertile than fish species, with females laying only one to seven eggs per litter [18]. Their long reproductive cycle and low fecundity have limited the breeding process and the development of large-scale Asian yellow pond turtle production. In our previous study, we evaluated the genetic diversity and relationships of four Asian yellow pond turtle populations based on the barcoding sequences [19], then the population from the southern group was established in 2005 to determine the productivity success of female turtles. Through the long-term observation and recording of the breeding and reproduction, we found significant individual differences in annual egg production, and the existence of such differences was confirmed by Cheng [20]. We also developed a multi-microsatellite system for paternity identification in the Asian yellow pond turtle [21].

The aim of this study was to measure the reproductive capacity of the Asian yellow pond turtle by counting the annual average number and variation patterns of offspring. We used paternity testing technology to evaluate the reproductive performance of each female turtle, and then the female turtles were divided into high and low reproductive capacity individuals. Our findings can provide the basis and materials for the construction of high-fertility groups and the cultivation of high-fertility varieties of the Asian yellow pond turtle.

## 2. Materials and Methods

### 2.1. Sampling

Turtles lay eggs by digging holes, one clutch at a time, with the same clutch being the offspring of the same mother. We first checked the spawning site daily for signs of turtle digging to determine if any turtles have laid eggs. We would incubate the eggs in the clutches and then mark and sample them. The female turtles in the experiment were reared in the same pond and bred in the same field (the pond area is 25 square metres), so the purpose of paternity testing was mainly to determine the mother of each clutch of eggs.

All parent turtles, including 89 females (average weight is 780.14 g) and 43 males (average weight is 823.13 g), were purchased from the Guangzhou Aquatic Thoroughbred Base of the Pearl River Fisheries Research Institute (Guangzhou, China) and marked by drilling a hole in the edge of the carapace and making stainless steel tags with numbers (No. 1–132, m and f denote mother and father, respectively. For example, maternal individual 1 is marked 1 m and paternal individual 1 is marked 1f). The Asian yellow pond turtle offspring were hatched in 2005 and artificially bred until 2016. Turtle eggs were collected and hatched from 2013 to 2016. Each hatch was first marked and then the individual and clutch numbers were recorded before they were allowed to breed together.

Nails are reproducible tissues, so sampling this tissue reduced any damage to the individuals. Nails from all parents and offspring were collected and stored in absolute ethyl alcohol. Total genomic DNA was isolated and purified from nail samples using the MicroElute Genomic DNA Kit (OMEGA bio-tek, Norcross, GA, USA). DNA integrity was checked by 1% agarose gel electrophoresis and DNA concentration was measured using a NanoQTM UV–vis Spectrophotometer (CapitalBio Corporation, Beijing, China), adjusted to 20 ng/μL and then stored at −20 °C. All animal experiments in this study were conducted according to the guidelines of the Pearl River Fisheries Research Institute, Chinese Academy of Fishery Sciences. All turtles used were treated humanely and ethically, and the experiments were approved by the Pearl River Fisheries Research Institute, Chinese Academy of Fishery Sciences.

### 2.2. Microsatellite Analysis

Two sets of multiplex PCR systems, each containing eight pairs of microsatellite primers, were used for genotype analysis. Microsatellites were amplified with fluorescence-labelled primers in 10 μL PCR reactions containing 1 μL nuclear DNA, 5 μL Multiplex PCR Master Mix (Applied Biosystems, Waltham, MA, USA), 2 μL of a 10 μmol/L forward and reverse primer mix (Sangon Biotech (Shanghai) Co., Ltd., Shanghai, China), 20 μmol/L fluorescent labelling and distilled water to 10 μL. Multiplex PCR was performed under the following conditions: initial denaturation at 94 °C for 5 min, followed by 26 cycles at 94 °C for 30 s, at 57 °C for 40 s, at 72 °C for 40 s, then 8 cycles at 94 °C for 30 s, at 53 °C for 40 s, at 72 °C for 30 s and a final extension of 10 min at 72 °C. PCR products were analyzed on an ABI3130 automated DNA Analyzer (Applied Biosystems USA) using the internal standard GeneScan 500 LIZ Size Standard (Applied Biosystems USA) [22].

Fragment lengths were scored using the Peak Scanner v1.0 (Applied Biosystems USA), which uses interpolation to derive size lengths. This scoring method achieved an accuracy of one base pair. Lengths and scores were manually confirmed. All samples with unexpected alleles were re-amplified and re-run for confirmation. If they were difficult to amplify in the multiplex, the loci of some samples were amplified individually for confirmation.

### 2.3. Population Analysis and Paternity Assessment

The genotypes of all parents and their offspring for four years were collected and scored as described above. To assess the suitability of our loci for parentage analysis, the expected heterozygosity and average exclusion probabilities were calculated using the ‘‘allele frequency’’ module in the program of CERVUS 3.0 [23,24]. Allele frequency analysis (including combined non-exclusion probabilities for first-, second-, and parental-pairs), simulation of parentage analysis and parentage analysis (parental pair sexes known) were performed with parentage assignment based on relaxed and strict LOD scores of 80% and 95% confidence, respectively. Allele number (*N_a_*), observed homozygosity (*H_o_*), expected heterozygosity (*H_e_*) and polymorphic information content (*PIC*) were also calculated. For each offspring tested, parentage was assigned to the most likely candidate parent with two predetermined confidence levels, referred to as relaxed (80%) and strict (95%) according to a previous study [21].

### 2.4. Reproductive Statistics Analysis

The data of spawning stock were collected and assessed using Statistical Package for the Social Sciences (SPSS) software (version 20). The one-way analysis of variance (ANOVA) test was used to assess whether there were any statistically significant differences in spawning stock between and within the years at a 95% confidence level.

## 3. Results

### 3.1. Reproductive Statistics

We recorded the offspring produced by the breeding population (86 females and 43 males) for four consecutive years from 2013 to 2016 (Table 1). A total of 1263 offspring were produced by the females. The number of offspring ranged from 285 to 352, and the mean individual number of offspring ranged from 3.31 to 4.09 with a standard deviation of 0.35. One-way analysis of variance showed that the differences in spawning indicators between years were not statistically significant (F = 0.941, *p* = 0.421) (Table S1).

### 3.2. Population Genetic Diversity Analysis

The genetic diversity analysis of female parents revealed that the number of alleles (*N_a_*) ranged from 4 to 24, and the average number of alleles was 12.188. The loci with the highest and lowest number of alleles were m13 and m3, respectively. The observed homozygosity (*H_o_*) ranged from 0.404 to 0.978 with a mean of 0.667. The expected homozygosity (*H_e_*) ranged from 0.379 to 0.932 with a mean of 0.743. Among these 16 microsatellite loci, most of the loci were highly polymorphic (0.500 < *PIC* < 1.000), except for m2, m3 and m4, which were moderately polymorphic (0.250 < *PIC* < 0.500). For male parental individuals, the number of alleles (*N_a_*) ranged from 5 to 19, and the average number of alleles was 11.688 (Table 2). The loci with the highest and lowest number of alleles were S10 and m3, m4, respectively. The observed homozygosity (*H_o_*) ranged from 0.419 to 0.907 with a mean of 0.690. The expected homozygosity (*H_e_*) ranged from 0.472 to 0.936 with a mean of 0.787. The expected homozygosity in male parental individuals was higher than that in female parental individuals, except for s22 loci. The highly polymorphic locus and the moderately polymorphic locus were similar to the female parent individuals.

As the reproductive situation varies each year, for the offspring, there were some changes in the number of alleles, polymorphic information content, observed heterozygosity and expected heterozygosity of each locus, but the average values of the offspring produced over the four years were not significantly different (Table S2).

### 3.3. Paternity Assessment

Paternity testing revealed that there are some differences between female offspring and their contribution to the population. The number of female offspring ranged from 0 to 15 in one season (Table S3). In total, 2 females (78 m, 106 m) produced no offspring in all four years and 41 females had offspring in each year (Table S3). Then, we set four as the standard for selecting females with good fecundity and calculated the total number of offspring, the average number of offspring and the proportion of offspring in the population (Table 3). These females with more than four offspring per year account for 62.11%~79.26% of the identified population with an average of 65.00%. Of note, there were seven females (124 m, 2 m, 27 m, 16 m, 43 m, 117 m, and 23 m) that produced more than five offspring in four consecutive years (Table 4 and Table S3).

### 3.4. Fertility Grouping

**Figure 1:**
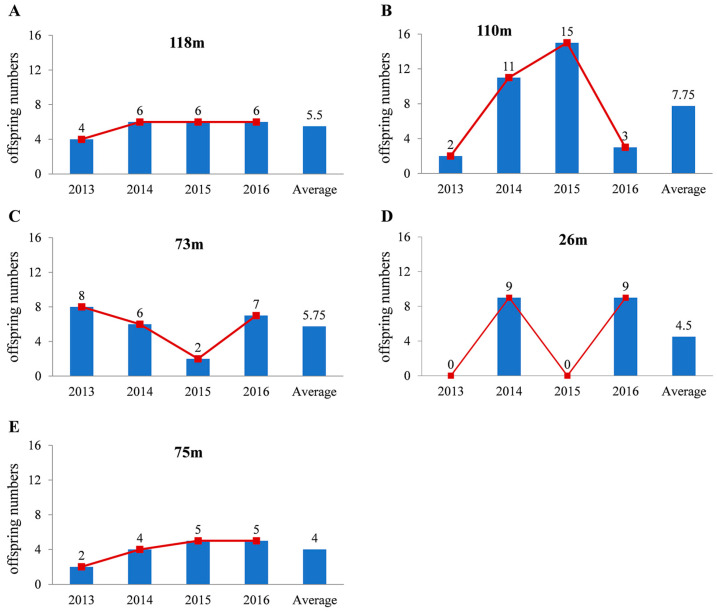
Female Asian yellow pond turtles are classified into 3 types based on the number of offspring they produce. More representative individuals of the 3 types are shown in (**A**) (stable type), (**B**–**D**) (undulating type) and (**E**) (levelling off type). The *x*-axis represents the year and the *y*-axis represents the number of offspring of female turtles.

Using the records of 86 females, we first analyzed the patterns of their generations over time; the number of female offspring can be divided into three types: stable, undulating and levelling off.

(1). Stable type. The variation in the annual number of offspring obtained for consecutive years was not significant. For example, female 118 m had four, six, six, and six offspring in the four years (Figure 1A).

(2). Undulating type. The general tendency of the progeny numbers changed with a sudden increase or decrease. The offspring number curve appears as an obvious peak. Female 110 m had 2, 11, 15, and 3 offspring in the four years (Figure 1B) and female 73 m had 8, 6, 2, and 7 offspring (Figure 1C). There is one notable characteristic of this species: continuous volatility. There are large changes between neighbouring years with a large number of peaks. Female 26 m had 0, 9, 0, and 9 offspring in the four years (Figure 1D).

(3). Leveling off type. The number of offspring leveled off with a continuous increase or decrease. Female 75 m had two, four, five, and five offspring in the four years (Figure 1E).

The females were divided into three types on the basis of offspring quantity and continuity, in order to provide the basis and materials for the construction of a good reproductive group.

(1). Low fertility. The average number of offspring was less than 3.5. For example, female 32 m had zero, one, zero, and zero offspring, with an average of 0.25.

(2). Medium fecundity. The average number of offspring ranged from 3.5 to 5. Female 109 m had four, eight, zero, and six offspring, with an average of 4.5.

(3). High fertility. The average number of offspring was over five. Female 118 m had 6 offspring for 3 years and 4 offspring in 2013, with an average of 5.5 offspring. Female 21 m had only 2 offspring in 2013, but 11, 11, and 7 offspring in the other years. The average number of litters was 7.75. The consistently high fecundity is a notable type that has more than five offspring in each year. For example, the 27 m female had eight, seven, seven, and five offspring in the four years.

## 4. Discussion

Turtles have distinctive breeding activities that create a long-term barrier to breeding selection. First, the female lays eggs in a burrow and then fills the burrow without protection or incubation. Some research has shown that many reptile species lay eggs in communal nests [25,26]. Some females lay eggs in close proximity to each other, making it difficult to collect eggs from the same female. In addition, turtles exhibit the phenomenon of multiple mating and multiple paternity, and females can lay eggs multiple times in a breeding season [14,27]. Together, these characteristics increase the difficulty of parental identification and population construction using traditional population selective breeding.

The Asian yellow pond turtle chosen for this study is a cultured population that does not interact with other populations, so immigration did not bias the paternity tests. The captive population had adequate food and nutrition, which excludes the effects of insufficient energy supply [28,29]. The closed environment also excluded any predators or pets that would destroy eggs, and eggs could be collected and recorded by nest on a daily basis. The eggs were hatched artificially, and this hatching technology has been used continuously for a long time [30]. By collecting whole offspring, the parentage of this population could be analyzed with consistency and accuracy. As there were no factors affecting egg hatching, the number of offspring was chosen as the standard for evaluating the fecundity of females. As all eggs were incubated under the same conditions, and the conditions were verified by previous experiments; individual differences were the main factors, excluding the preference errors due to external factors.

Females are sexually mature until they are 5 years old, and their fecundity goes through growth, stabilization and decline [7]. Compared to turtles with low and medium fecundity, females with more offspring are more suitable for artificial culture with a long-term pre-breeding period. Some females with significant fecundity changes also have the disadvantage of not being able to determine their number of offspring, as their contingency is detrimental to seed supply and standardized breeding. The females in the experimental population were 11 years old and the average number of offspring was increasing (Table 1), so the sudden decrease in offspring number was caused by individual factors under the same rearing conditions.

Microsatellite technology was used in the paternity testing and genetic research [31,32], so the results are highly realistic and accurate. Multiple mating, multiple paternity and sperm preservation make it difficult to construct a family or half-sibling family [33,34]. Therefore, we tried to select females with good fertility directly according to the population perspective. By collecting females with high or low fertility, this population could be used for research on reproductive mechanisms and marker-assisted selection (MAS) of the Asian yellow pond turtle. In this study, we found that seven females (124 m, 2 m, 27 m, 16 m, 43 m, 117 m, and 23 m) had more than five offspring in each year, accounting for 16.94% of the total offspring (Table 3). They have consistently high fecundity, which is good for meeting market needs with accurate prediction and control. Some females had little or no offspring during the 4 years, while others had a high reproductive capacity. We also found that the number of offspring per female increased with the maternal plastron length rather than age [35]. These results suggest that paternity testing can be used to analyze differences in female fecundity, select females with high and stable fecundity and eliminate females with low fecundity, thus serving as the next step in the selection of highly fecund turtles (Table S3).

We also assessed population genetic diversity based on polymorphism information content and heterozygosity. In this study, the parental *H_o_* was 0.690 and 0.667, and the *H_e_* was 0.787 and 0.743 (Table 2). These data show high genetic diversity and are similar to the RAPD results of two *Mauremys mutica* populations [36]. Furthermore, female 110 m had 15 offspring in 2015, while some females had few or no offspring. This phenomenon could result in a significant preference for some gene frequencies in this batch of offspring. This reproductive characteristic allows the dominant individual with a stronger reproductive capacity to transmit its genes to more offspring and help the small population to increase rapidly, but it can also cause degeneration due to the accumulation of some disadvantageous factors, the loss of some advantageous factors or increasing the possibility of inbreeding. Therefore, when selecting parents for cultured stocks, their genetic background information should be obtained in order to maintain high genetic diversity. Considering that males can also influence the number of offspring [37], the identification data for four consecutive years are useful to evaluate the genetic contribution of males in further studies. Furthermore, this experiment will be continued to test whether the offspring of highly fertile individuals have inherited this high-fertility trait.

In summary, we used microsatellite markers to analyze genetic diversity and used parentage assignment to determine the offspring of each mother in the population over four years. In addition, progeny testing was used to select breeders with high or low fertility traits. These results will provide useful material for Asian yellow pond turtle breeding research.

## 5. Conclusions

In this study, we measured the reproductive capacity of the Asian yellow pond turtle by counting the annual average number and variation patterns of offspring. We also evaluated the reproductive performance of each female turtle based on paternity testing technology, and then the female turtles were divided into high and low reproductive capacity individuals. Our findings can provide the basis and materials for the construction of high-fertility groups and the cultivation of high-fertility varieties of the Asian yellow pond turtle.

## Figures and Tables

**Table 1:** Four-year reproductive results of the Asian yellow pond turtle females.

Year	Female Numbers	Offspring Numbers	Average Individual Number of Offspring
2013	86	285	3.31
2014	86	297	3.45
2015	86	352	4.09
2016	86	329	3.83
Annual Average Number	86	315.75	3.67

**Table 2:** Genetic diversity of the parents of the Asian yellow pond turtle in the population.

Locus	Female				Male			
	*N_a_*	*H_o_*	*H_e_*	*PIC*	*N_a_*	*H_o_*	*H_e_*	*PIC*
s8	8	0.614	0.757	0.715	8	0.651	0.788	0.746
m13	24	0.978	0.92	0.909	19	0.86	0.936	0.920
m1	12	0.629	0.668	0.644	10	0.698	0.786	0.753
m7	22	0.632	0.886	0.872	18	0.698	0.915	0.898
m2	6	0.404	0.379	0.349	6	0.558	0.538	0.482
s10	19	0.775	0.932	0.922	22	0.744	0.936	0.920
m3	4	0.416	0.407	0.347	5	0.419	0.472	0.421
m4	5	0.416	0.512	0.457	5	0.442	0.556	0.494
s3	8	0.708	0.745	0.702	7	0.605	0.769	0.722
s16	13	0.726	0.843	0.820	13	0.805	0.854	0.824
s11	15	0.888	0.873	0.855	14	0.907	0.896	0.875
s29	11	0.64	0.821	0.791	11	0.721	0.847	0.817
s22	12	0.798	0.869	0.849	12	0.791	0.859	0.832
s5	9	0.448	0.576	0.551	10	0.512	0.702	0.669
s23	17	0.798	0.888	0.873	17	0.837	0.921	0.904
s27	10	0.798	0.806	0.775	10	0.791	0.824	0.794
Average	12.188	0.667	0.743	0.714	11.688	0.690	0.787	0.754

**Table 3:** The characteristics of the Asian yellow pond turtle females with more than 4 offspring every year.

	2013	2014	2015	2016	Average of 4 Years
The female numbers with more than 4 offspring	22	34	34	37	33
Total offspring number	177	226	279	258	205.25
Average offspring number	8.05	6.65	8.21	6.97	6.22
The ratio of offspring in the population (%)	62.11	73.62	79.26	78.41	65.00

**Table 4:** The reproductive results of 7 females (124 m, 2 m, 27 m, 16 m, 43 m, 117 m and 23 m).

	2013	2014	2015	2016
total offspring number	57	48	57	52
average offspring number	8.14	6.86	8.14	7.43
the ratio in the population (%)	19.26	15.64	16.19	15.81

## Data Availability

The data presented in this study are available in the article and in the Supplementary Material.

## Supplementary Materials

The following supporting information can be downloaded at: https://www.mdpi.com/article/10.3390/ani14030479/s1, Table S1: One-way analysis of variance for four-year egg production in the Asian yellow pond turtle; Table S2: Genetic diversity of the offspring of the Asian yellow pond turtle in the population; Table S3: The offspring number of each female over four years.
