# Supplementary material for: Assessing and Screening of Female Fertility in Artificially Bred Asian Yellow Pond Turtles (Mauremys mutica) Based on Parentage Assignment
Source: Animals (Basel). 2024 Feb 1;14(3):479. doi: 10.3390/ani14030479 (PMC10854808; doi:10.3390/ani14030479)
Supplement: Supplementary file 1 [file animals-14-00479-s001.zip › animals-2803043-supplementary.pdf]

Table S1: One-way analysis of variance for four-year egg production in the Asian yellow pond turtle.

	Sum of squares	Degrees of freedom	Mean square	F crit	Significance
Within years	32.404	3	10.801	0.941	0.421
Between years	3903.477	340	11.481		
Total	3935.881	343			

Table S2. Genetic diversity of the offspring of the Asian yellow pond turtle in the population.

	**Offspring of 2013**				**Offspring of 2014**				**Offspring of 2015**				**Offspring of 2016**			
Locus	N_a	H_o	H_e	*PIC*	N_a	H_o	H_e	*PIC*	N_a	H_o	H_e	*PIC*	N_a	H_o	H_e	*PIC*
s8	10	0.568	0.782	0.754	9	0.605	0.761	0.720	8	0.558	0.770	0.731	11	0.699	0.799	0.770
m13	24	0.912	0.918	0.911	24	0.960	0.929	0.923	24	0.929	0.932	0.927	27	0.932	0.936	0.931
m1	14	0.750	0.782	0.763	12	0.735	0.755	0.733	13	0.739	0.747	0.725	14	0.817	0.767	0.749
m7	19	0.706	0.906	0.897	21	0.681	0.891	0.881	22	0.815	0.907	0.898	27	0.860	0.910	0.903
m2	9	0.601	0.591	0.538	7	0.482	0.485	0.437	8	0.474	0.496	0.456	9	0.648	0.568	0.530
s10	20	0.639	0.926	0.920	21	0.732	0.933	0.927	21	0.861	0.915	0.907	24	0.902	0.932	0.927
m3	5	0.422	0.420	0.378	5	0.434	0.396	0.350	5	0.358	0.405	0.364	5	0.598	0.527	0.485
m4	5	0.341	0.593	0.551	5	0.397	0.586	0.532	5	0.472	0.578	0.530	6	0.570	0.624	0.566
s3	8	0.750	0.763	0.725	8	0.717	0.764	0.728	8	0.722	0.744	0.704	9	0.738	0.768	0.733
s16	13	0.587	0.821	0.797	13	0.670	0.857	0.840	14	0.731	0.842	0.824	14	0.767	0.853	0.837
s11	17	0.855	0.875	0.860	17	0.887	0.888	0.875	19	0.885	0.890	0.878	18	0.947	0.897	0.887
s29	14	0.828	0.847	0.829	12	0.775	0.821	0.797	13	0.813	0.832	0.810	13	0.821	0.818	0.796

s22	13	0.814	0.848	0.828	12	0.745	0.846	0.826	10	0.764	0.836	0.815	14	0.854	0.857	0.840
s5	10	0.530	0.591	0.569	11	0.520	0.623	0.601	12	0.590	0.684	0.658	11	0.580	0.650	0.629
s23	18	0.916	0.901	0.891	17	0.834	0.896	0.885	17	0.895	0.904	0.895	21	0.858	0.907	0.899
s27	10	0.834	0.830	0.807	9	0.868	0.803	0.776	9	0.827	0.776	0.745	11	0.836	0.802	0.776
Average	13.063	0.691	0.775	0.751	12.688	0.690	0.765	0.739	13	0.714	0.766	0.742	14.625	0.777	0.788	0.766

Table S3. The offspring number of each female over four years.

**Year**	**Offspring numbers**	**Number of females**	**Female turtle No.**												
201	0	13	102m	105m	111m	112m	24m	26m	28m	32m	34m	39m	40m	59m	69m
3	1	13	113m	120m	121m	13m	15m	1m	36m	44m	4m	51m	57m	60m	62m
	2	11	110m	115m	126m	19m	21m	29m	48m	53m	75m	80m	9m		
	3	11	108m	116m	11m	127m	129m	31m	41m	46m	50m	67m	82m		
	4	9	101m	109m	118m	12m	131m	45m	70m	72m	83m				
	5	3	42m	92m	94m										
	6	2	104m	33m											
	7	2	124m	18m											
	8	8	130m	16m	27m	2m	43m	73m	88m	89m					
	9	3	103m	117m	23m										
	10	3	17m	99m	9m										
	12	1	38m												
	13	1	114m												
201	0	11	112m	24m	40m	78m	97m	106m	120m	15m	36m	44m	4m		

4	1	11	102m	105m	32m	13m	62m	68m	74m	48m	131m	42m	33m	
	2	8	111m	121m	60m	29m	50m	83m	92m	99m				
	3	8	28m	39m	46m	101m	45m	70m	18m	88m				
	4	5	59m	75m	11m	130m	89m							
	5	10	34m	69m	84m	115m	108m	16m	2m	43m	103m	17m		
	6	12	113m	1m	126m	80m	9m	67m	118m	12m	72m	104m	73m	23m
	7	4	79m	116m	82m	27m								
	8	2	53m	109m										
	9	3	26m	117m	38m									
	11	3	110m	21m	124m									
201	0	9	78m	106m	15m	44m	4m	57m	19m	31m	32m			
5	1	8	40m	102m	111m	92m	39m	45m	11m	104m				
	2	10	36m	129m	74m	131m	83m	59m	34m	69m	80m	73m		
	3	7	24m	97m	101m	115m	103m	113m	79m					
	4	6	127m	114m	105m	28m	46m	12m						
	5	6	112m	41m	68m	75m	126m	117m						
	6	6	120m	51m	94m	1m	118m	124m						
	7	7	99m	70m	88m	108m	43m	67m	27m					
	8	3	50m	130m	89m									
	9	1	2m											
	10	2	29m	16m										
	11	4	18m	53m	38m	21m								
	12	1	82m											
	13	2	23m	116m										
	14	1	48m											
	15	1	110m											

2016	0	17	105m	106m	113m	129m	13m	15m	24m	32m	36m	40m	42m	112m	57m	67m	68m	78m	83m
	1	8	45m	115m	130m	1m	28m	39m	4m	84m									
	2	13	101m	104m	108m	111m	114m	11m	127m	131m	29m	48m	72m	94m	9m				
	3	7	110m	120m	19m	31m	34m	92m	38m										
	4	4	50m	53m	60m	74m													
	5	11	102m	16m	18m	27m	2m	46m	51m	59m	75m	82m	99m						
	6	10	103m	109m	117m	118m	124m	126m	44m	69m	89m	97m							
	7	5	21m	70m	73m	80m	62m												
	8	4	33m	41m	79m	88m													
	9	3	121m	17m	26m														
	11	2	12m	43m															
	13	1	116m																
	14	1	23m																

*The meaning of “m” represents maternal individual.
